# Cyberattack on the NHLS Network: Highlighting the crucial role of laboratory medicine

**DOI:** 10.4102/jcmsa.v2i1.108

**Published:** 2024-08-23

**Authors:** Tahir S. Pillay

**Affiliations:** 1 Department of Chemical Pathology, Faculty of Health Sciences, University of Pretoria, Pretoria, South Africa; 2 Tshwane Academic Division, National Health Laboratory Service, Pretoria, South Africa; 3 Division of Chemical Pathology, University of Cape Town, Cape Town, South Africa

**Keywords:** NHLS, ransomware, cyberattack, network, internet, pathology, diagnosis, laboratory medicine

## Abstract

The ransomware cyberattack on the National Health Laboratory Service (NHLS) network highlighted the indispensable role of laboratory medicine in the healthcare system. This incident severely disrupted the processing and release of patient samples, underscoring the critical yet often underappreciated contributions of laboratory medicine. Laboratory professionals operate behind the scenes, providing data crucial for medical decisions, yet they frequently face challenges such as resource allocation, limited public awareness, and professional recognition. In order to address the ‘Cinderella specialty’ perception of laboratory medicine, it is essential to enhance visibility, education, and resource allocation. Governments and politicians can play a pivotal role in this transformation by increasing funding for laboratory infrastructure and technology, reinforcing laboratory medicine in medical curricula, launching public awareness campaigns, and supporting workforce development. In addition, fostering innovation through research grants and policy advocacy can further elevate the status of laboratory medicine. By recognising and addressing these issues, the healthcare system can ensure that the contributions of laboratory professionals are fully appreciated, leading to improved patient care and health outcomes.

Many of the Colleges of Medicine, South Africa (CMSA) members will have, by now, experienced the impact of the June 2024 ransomware cyberattack on the National Health Laboratory Service (NHLS) computer network resulting in the paralysis of processing of patient samples for analysis and the release of laboratory results. These rely heavily on electronic processing and transmission from the time of specimen receipt to the point of reporting. A paralysis of the network severely compromises the ability of doctors to make clinical decisions in the public healthcare sector.

In common with the coronavirus disease 2019 (COVID-19) pandemic,^1,2^ this crisis has underscored the indispensable role of laboratory medicine in the healthcare system. Despite its essential nature, laboratory medicine is often perceived as a ‘Cinderella specialty’ – vital, yet underappreciated. What are the key reasons for this perception?

## Work behind-the-scenes

Laboratory medicine operates largely out of public view.^3^ Unlike clinicians and surgeons who interact directly with patients, laboratory professionals work in the background, analysing samples and generating crucial data for diagnoses and treatments. This invisibility can lead to their role being undervalued.

## Underestimation of impact

Approximately, 70% of medical decisions are based on laboratory results,^4,5^ yet the public and even many healthcare professionals often overlook^3,6^ this significant contribution to patient care.

## Resource allocation

Laboratory departments and services frequently receive less funding and fewer resources compared to other clinical departments, resulting in outdated equipment, staffing shortages, and limited opportunities for professional development.^7,8,9^ *(Even within the CMSA, diverse laboratory specialities are lumped into an overarching College of Pathologists underscoring the perceptions alluded to above).*

## Education and awareness

Medical education typically emphasises clinical skills and direct patient care, leaving laboratory medicine less emphasised. Consequently, there is a general lack of awareness about its scope and importance.

## Professional recognition

Laboratory professionals, including medical laboratory scientists and pathologists, often experience lower recognition and status compared to clinical counterparts, affecting morale and the appeal of the speciality to new entrants.^10^

## Perceived routine nature

Laboratory work is sometimes seen as routine and monotonous. This perception overlooks the complexity and critical thinking required in various pathology disciplines such as chemical pathology, haematology, anatomical pathology, microbiology, genetics, virology, and immunology.

## Public perception

The general public often has a limited understanding of what laboratory medicine entails, typically envisioning doctors and nurses rather than the laboratories that play a crucial role in diagnosing diseases and monitoring treatment efficacy.

## Historical context

Historically, laboratory medicine has evolved from a support service to a specialised field. However, the historical view of it as a support function still lingers, contributing to its ‘Cinderella’ status. One need only hark back to the era of the ‘barber surgeon’. Barber-surgeons were so named because, in the medieval period, they performed a dual role as barbers and surgeons. The origin of this dual profession can be traced back to a time when surgery was considered a manual craft rather than a scholarly medical practice. Internal medicine physicians of the time, who were perceived to be more academic and focused on diagnosis, viewed surgery as a lower-status occupation. Physicians, who studied at universities and were part of a more elite and scholarly medical profession, often looked down upon the practical and manual nature of surgery. In the 18th and 19th centuries, surgery became more respected and formally integrated into medical education and practice. A similar paradigm shift needs to take place with pathology and laboratory medicine.

## Addressing the Cinderella perception

In order to address this perception, it is essential to:

- Increase the visibility of the contributions of laboratory professionals to patient care.
- Enhance education and awareness about the importance of laboratory medicine.
- Advocate for better resource allocation and professional recognition.
- Highlight innovations and advances within the field, such as molecular diagnostics and personalised medicine.

By promoting a better understanding of the pivotal role laboratory medicine plays in healthcare, its status can be elevated from a ‘Cinderella specialty’ to one that is fully recognised and valued for its contributions.

## Role of governments and politicians

Governments and politicians can play a crucial role in elevating the status and recognition of laboratory medicine through various actions. Governments should take the following steps:

### Increase funding and resources

- Allocate more budget for laboratory infrastructure, equipment, and technology, including network infrastructure, to ensure laboratories are well-equipped and protected from cyberattacks. The NHLS is funded separately from the Department of Health and directly from Treasury.
- Provide research grants to foster innovation and new diagnostic methods.

### Enhance education and training

- Facilitate the integration of laboratory medicine more comprehensively by universities into medical and health science curricula by funding mechanisms similar to the Clinical Training Grants (CTG) and Health Professions Training and Development grants (HPTDG).
- Offer scholarships, fellowships, and training programmes to attract talented individuals to the field.
- Encourage and facilitate the role of the private sector in providing and funding training. In South Africa, the private sector absorbs the newly qualified specialists, but provides no funding for training.

### Public awareness campaigns

- Launch campaigns highlighting the vital role of laboratory medicine in healthcare.
- Establish awards and recognition programmes for outstanding contributions in the field.

### Policy and regulation

- Implement and enforce high standards for laboratory operations to ensure quality and reliability of diagnostic services. The South African National Accreditation System (SANAS) accreditation system has an extensive footprint in South Africa, in the NHLS and in the private sector.
- Support professional accreditation and certification programmes for laboratory personnel.

### Support workforce development

- Promote job creation in the laboratory sector and facilitate continuous professional development opportunities through workshops, seminars, and online courses.

### Integrate laboratory medicine into public health initiatives

- Enhance the role of laboratory medicine in national disease surveillance programmes and engage laboratory professionals in public health campaigns.^4^ In South Africa, this is well established with the National Institute of Communicable Diseases (NICD).

### Collaboration with professional bodies

- Partner with professional bodies and organisations in laboratory medicine to develop supportive policies and initiatives.
- Regularly consult laboratory professionals when drafting healthcare policies.

### Promote innovation and technology adoption

- Provide incentives for laboratories to adopt cutting-edge technologies and innovative practices.
- Support the integration of digital health solutions with laboratory information systems to streamline workflows and enhance patient care.

### Health policy advocacy

- Advocate for legislation supporting the development and sustainability of laboratory medicine.
- Include laboratory medicine experts in public health policy development to recognise their critical role in disease prevention and management.^11^

By taking these steps, governments and politicians can help transform laboratory medicine from a ‘Cinderella specialty’ into a highly recognised and valued component of the healthcare system, ensuring that its contributions are fully appreciated and supported.
